# A Comparable icELISA and Lateral Flow Immunoassay for the Sensitive and Rapid Detection of 4,4′-Dinitrocarbanilide in Chicken

**DOI:** 10.3390/toxics11070628

**Published:** 2023-07-20

**Authors:** Qianxin Liang, Chen Chen, Wenqing Xu, Ning Zhang, Jielin Yang, Wei Song, Huimei Cai, Ruyan Hou, Hongfang Li, Xiya Zhang

**Affiliations:** 1 Animal-Derived Food Safety Innovation Team, College of Tea and Food Science & Technology, Anhui Agricultural University, Hefei 230036, China; 22721093@stu.ahau.edu.cn (Q.L.); 22721096@stu.ahau.edu.cn (C.C.); xwq18715604402@163.com (W.X.); chm@ahau.edu.cn (H.C.); hry@ahau.edu.cn (R.H.); 2 Hefei Customs District Technical Center, Anhui Key Lab of Analysis and Detection for Food Safety, Hefei 230022, China; songwei1768@163.com; 3 College of Food Science and Technology, Henan Agricultural University, Zhengzhou 450002, China; zhangxiya@henau.edu.cn

**Keywords:** icELISA, lateral flow immunoassay, 4,4′-dinitrocarbanilide, rapid detection, chicken

## Abstract

4,4′-dinitrocarbanilide (DNC) is a key component and marker residue of nicarbazin, which forms residues in edible tissue and then causes nephrotoxicity and hepatotoxicity in humans if used excessively. To simplify sample preparation and monitor the DNC rapidly and accurately, a comparable icELISA and lateral flow immunoassay (LFIA) was developed in this study. Briefly, the reaction parameters were explored for improving the sensitivity of icELISA and LFIA. Under the optimal conditions, methanol was selected as the extracting solvent for DNC in chicken, and 20- and 10-fold dilutions of sample extraction eliminated the matrix effect for icELISA and LFIA, separately. After sample pretreatment, the analysis properties of icELISA and LFIA were compared. The limit of detection of icELISA for DNC was 0.8 μg/kg, and the visual and quantitative limits of detection of LFIA were 8 and 2.5 μg/kg. Compared with icELISA, LFIA showed lower sensitivity but obvious advantages in terms of matrix tolerance and detection time (within 15 min). The sensitivity, specificity, and accuracy of the developed assays satisfied the detection requirement even if using simple sample pretreatment. This comparable icELISA and LFIA provided mutual verifiability methods for the accurate detection of DNC in chicken.

## 1. Introduction

Coccidiosis is a common parasitic disease in poultry, which has negative impacts on the absorption of nutrients and feed conversion, and leads to loss of weight and even death of poultry animals [1]. At present, anticoccidial drugs are the most effective approach to control coccidiosis, and among these, nicarbazin belongs to chemical synthesis drugs and is frequently used worldwide for its advantages of higher activity and lower drug resistance [2]. Nicarbazin consists of an equimolar complex of 4,4′-dinitrocarbanilide (DNC, Figure S1) and 2-hydroxy-4,6-dinitrocarbanilide (HDP). In detail, DNC actually possesses anticoccidial activity and is metabolized slowly. Once it has formed a residue in animal-derived foods, DNC causes nephrotoxicity, hepatotoxicity, and reproductive toxicity to humans [3]. Meanwhile, HDP plays the role of synergist that promotes the absorption of DNC in the body, which metabolizes rapidly and is then excreted via urine [4]. Thus, DNC is internationally recognized as a residue marker for nicarbazin. Many countries, e.g., the United States, China, New Zealand, and so on, have established a maximum residue limit for DNC in animal-derived food for preventing the abuse of DNC and protecting consumer health. For example, the maximum residue limit has been set to 200 μg/kg for DNC in chicken edible tissue and 0 μg/kg in egg samples, respectively [5,6]. Hence, quantitative, sensitive, and accurate analytical methods are greatly needed to monitor DNC residues and ensure food safety.

At present, the analytical methods for DNC in chicken, liver, and eggs mainly depend on instrumental analysis methods, including high-performance liquid chromatography, liquid chromatography–tandem mass spectrometry, and gas chromatography [7]. Although these methods are accurate, they tend to be complex in terms of sample preparation, and expensive and laborious in the detection procedure [8]. However, the analysis pattern of rapid detection is developing to be the alternative method for the rapid and immediate screening of DNC residues in samples [9]. Therefore, rapid, specific, and accurate analysis methods are in great demand for the detection of DNC.

Immunoassays, based on the specific recognition of antibodies and antigens, are consistently considered the traditional rapid detection methods with high specificity and satisfactory accuracy [10]. Currently, the indirect competitive (icELISA) and lateral flow immunoassays (LFIA) are frequently used methods in many areas, such as clinical diagnosis, environment, and food monitoring [11]. The corresponding rapid test kits have been widely produced and applied worldwide, and the well-known manufacturers producing the kits include Roche, R-Biopharm, and Abbott laboratories. Generally, icELISA can realize the high-throughput screening of samples (at least 40 samples). Compared with icELISA, LFIA is based on gold nanoparticles (AuNPs) and possesses the advantage of a short detection time (within 10 min) [12]. Thus far, a few icELISA and LFIA have been reported for the detection of DNC [9,13]. Moreover, the sample pretreatment varies for different studies, which hardly provides a standard reference for other researchers. There are two main approaches reported for sample pretreatment for DNC in chicken. The first approach is complex and time-consuming, including many procedures, e.g., extraction, concentration, purification, concentration, dilution, and detection [4]. These complicated steps are not suitable for the on-site detection of DNC. The second approach includes three steps, namely extraction, dilution, and detection [14]. Despite the procedure being simplified, this had to be compensated for by reducing the sensitivity of the analysis method because of the higher dilution fold. Therefore, a more sensitive analysis method is essential in order to shorten the detection period and achieve rapid detection.

In this study, we explored the influence mechanism by which reaction parameters (antigen and antibody concentrations, pH values, etc.) affect the sensitivity of the analysis method. We further evaluated the matrix effect of chicken and developed a sample pretreatment method for DNC using extraction and dilution procedures, which reduced the analysis time. Under optimal conditions, icELISA and LFIA were established with improved sensitivity and could satisfy the detection requirements of the maximum residue limit. Meanwhile, we compared the detection parameters (limit of detection, specificity, and accuracy) between icELISA and LFIA. This study provides two comparable analysis methods for the rapid, sensitive, and accurate detection of DNC in food samples.

## 2. Materials and Methods

### 2.1. Reagents and Instruments

Bovine serum albumin (BSA), tetramethylbenzidine, and horseradish peroxidase were obtained from Merck (Merck, Darmstadt, Germany). Goat anti-mouse IgG was purchased from Shanghai Kinbio Tech. Co., Ltd. (Shanghai, China). Nicarbazin was obtained from Biovet JSC (Peshtera, Bulgaria). Chloramphenicol, trimethoprim, and sulphanilamide were purchased from J&K Scientific Ltd. (Beijing, China). Coating antigen and monoclonal antibody 14D2 were prepared and provided by the Xiya Zhang research team at Henan Agricultural University. Trisodium citrate, aurichlorohydric acid, K_2_CO_3_, methanol, acetonitrile, and other chemical reagents were provided by Sinopharm Chemical Reagents Co., Ltd. (Shanghai, China). The chicken sample was purchased from Darunfa Market (Hefei, China) and was verified to be negative by liquid chromatography–mass spectrometry.

Nitrocellulose membrane (Millipore 135) was obtained from Millipore AG (Millipore AG, Zug, Switzerland). A 96-well microplate was purchased from Yunpeng Technology Development Co., Ltd. (Xiamen, China). Blood filtration membrane, polyvinyl chloride plates, and absorbent paper were purchased from Shanghai Kinbio Tech. Co., Ltd. (Shanghai, China). A Milli-Q Ultrapure water meter was obtained from Millipore (Massachusetts, USA). An HQ-6-Ⅱ vortex mixer was bought from Beijing North Tongzheng Biotechnology Development Co., Ltd. (Beijing, China).

### 2.2. Development and Optimization of icELISA for DNC in Buffer Solution

#### 2.2.1. Experimental Steps of icELISA

**Scheme 1:**
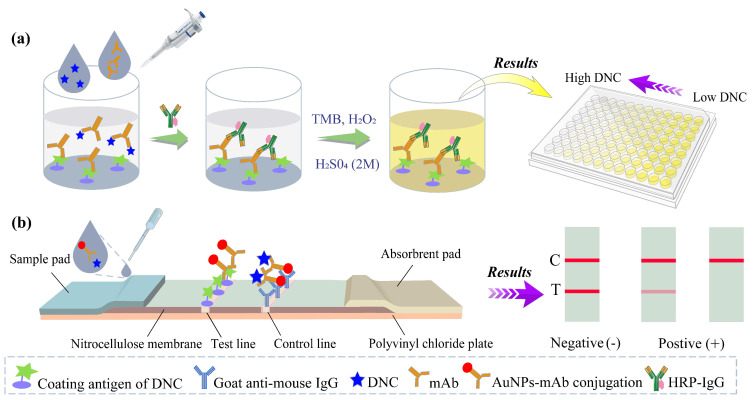
The schematic illustration of (**a**) icELISA and (**b**) LFIA for the detection of DNC. TMB indicates tetramethylbenzidine. T and C indicate the test line and control line.

The ELISA plates were first coated with coating antigen (100 μL/well) which was diluted with carbonate buffer solution (pH 9.6) and then incubated at 37 °C for 2 h. A washing solution (280 μL/well) was added, and the ELISA plates were washed 3 times. Subsequently, a blocking buffer (150 μL/well) was added and incubated for 1 h at 37 °C. The ELISA plates were washed by washing buffer three times and stored in the refrigerator before use. The standard solutions of DNC (or sample extraction, 50 μL/well) and mAb (50 μL/well) were added to the ELISA plates and incubated for 30 min at 37 °C. After three washings, horseradish peroxidase-labeled IgG (HRP-IgG) was added to the ELISA plates, incubating for 30 min at 37 °C. Then, tetramethylbenzidine that acted as the substrate (100 μL/well) of horseradish peroxidase was added into wells and incubated for 15 min at 37 °C after washing. Finally, H_2_SO_4_ (50 μL/well, 2 M) was added to stop the reaction, and the OD values at 450 nm were measured. The schematic illustration of icELISA for DNC detection is shown in Scheme 1a.

#### 2.2.2. Optimization of icELISA

To improve the sensitivity of icELISA, we optimized several crucial factors during the specific recognition of the antibody and antigen, including antigen and antibody concentrations, pH values and ion strength of buffer solution, and organic solvent tolerance.

A checkboard assay was preliminarily adopted to evaluate the antigen and antibody concentrations. The antigen concentrations were set as 0.4, 0.2, 0.1, and 0.05 μg/mL, and the monoclonal antibody (mAb) 14D2 ones were 6.25, 12.5, 25, 50, 100, and 200 ng/mL, respectively. The DNC concentrations were 0 and 0.5 ng/mL when exploring antigen and antibody pairs at different concentrations.

Phosphate buffer solutions (PBS) at different pH values (6.0, 6.5, 7.0, 7.4, and 8.0) and at diverse ion strengths (0.07, 0.14, 0.2, 0.5, and 1.0 mol/L) were chosen as the dilution buffers of the antigen and antibody. Simultaneously, the DNC concentrations were prepared to yield standard curves, with the eight dilution gradients of 0, 0.01, 0.03, 0.09, 0.27, 0.81, 2.43, and 7.29 ng/mL.

Two commonly used organic solvents (methanol and acetonitrile) were used as the dilution buffer of the DNC, then the standard curves were constructed. The concentrations of methanol and acetonitrile were 0%, 5%, 10%, 15%, and 20% (in the optimal PBS). Under the optimal conditions, the standard curve of DNC was fitted in the buffer solution using a four-parameter equation (Origin Software 2019). The maximal inhibition concentration (IC_50_) of ELISA was calculated.

### 2.3. Development and Optimization of LFIA for DNC in Buffer Solution

#### 2.3.1. Synthesis of Gold Nanoparticles

AuNPs were synthesized according to previous research [15]. Briefly, 1 L of the ultrapure water was heated to 100 °C, and then 40 mL of 1% HAuCl_4_·4H_2_O (*w*/*v*) was added under a magnetic stirrer until heated to 100 °C. Then, 50 mL trisodium citrate solution (1%, *w*/*v*) was added into the mixture solution and heated for 30 min, until the color of the mixture turned to claret. Finally, the AuNPs were cooled and stored at room temperature. AuNPs were characterized by UV–visible spectroscopy, transmission electron microscopy, and dynamic light scattering.

#### 2.3.2. Preparation of AuNPs–mAb Conjugate

AuNPs (1 mL) were placed into a centrifuge tube (1.5 mL), followed by adding K_2_CO_3_ (0.1 M) for adjusting the pH values of AuNPs. The mAb 14D2, diluted with pure water, was conjugated to AuNPs via electrostatic adsorption [16]. After incubation for 20 min, BSA solution (40 µL, 10%) was added to the AuNPs to block the unbound site, and then the mixture solution was incubated for 10 min. Afterward, the AuNPs–mAb conjugate was purified and separated using centrifugation (13,201 g, 15 min). Finally, the AuNPs–mAb conjugate was dissolved in PBS (20 mM, pH 7.4, 200 µL) and stored in a fridge (4 °C).

#### 2.3.3. Assembling of Lateral Flow Test Strips

First, goat anti-mouse IgG and coating antigen were coated onto the nitrocellulose membrane as the control (C) line and test (T) line, respectively. Then, the nitrocellulose membrane was incubated in an oven at 45 °C for 2 h. Subsequently, the nitrocellulose membrane, sample pad, and absorbent paper were successively pasted onto the polyvinyl chloride plates to assemble the test strips. The polyvinyl chloride plates were cut into test strips with a width of 3.18 mm by a cutting machine.

The detection process for the lateral flow immunoassay was as follows: AuNPs–mAb conjugates (4 µL) were placed onto the microplate, and then DNC solution (or sample solution, 145 µL) was added and mixed thoroughly for 5 min. The test strip was inserted into the mixture solution, and then, the test strip was observed by the naked eye and measured by Image-J software after 5 min of incubation. The schematic illustration of LFIA for DNC detection is shown in Scheme 1b.

#### 2.3.4. Optimization of LFIA Parameters

Several key parameters for LFIA were optimized, such as pH values of AuNPs, antibody and antigen concentrations, dilution buffer types of antibodies, and the amount of AuNPs–mAb conjugate. In detail, K_2_CO_3_ solution (0.1 M) at different volumes (10, 20, 30, 40, 60, and 80 µL) was added to adjust the pH values of AuNPs. The concentrations of mAb 14D2 were set as 3, 5, 7, and 9 µg/mL, and then AuNPs were labeled with the antibody. The mAb 14D2 was diluted with three buffers, PBS (20 mM, pH 7.4), Tris-HCl buffer (20 mM, pH 7.4), and 1% BSA buffer (in PBS, 20 mM, pH 7.4). Then, the volumes of AuNPs–mAb conjugate were 2, 3, 4, and 5 µL, respectively. Finally, the antigen at different concentrations (500, 250, 167, 125, 83, and 62.5 µg/mL) was explored. These parameters were optimized using monofactor analysis [17]. Under optimal conditions, the standard curve of DNC was generated using LFIA.

### 2.4. Sample Pretreatment of Chicken

DNC-negative chicken (1 g) was homogenized and extracted with methanol (2 mL). The mixture was vortexed for 10 min as well as centrifuged at 9168 g for 10 min, and the supernatant was collected. Subsequently, the extract solution was diluted to different folds with optimal buffer solution and was then used to generate matrix standard curves.

### 2.5. Detection Properties of icELISA and LFIA

#### 2.5.1. Limit of Detection

The IC_10_ of the matrix standard curve that could eliminate the matrix effect was calculated as the limit of detection for the ELISA [18]. For LFIA, the cut-off value served as the visual limit of detection, and the IC_10_ of the matrix standard curve acted as the quantitative limit of detection.

#### 2.5.2. Specificity

Non-target veterinary medicines were spiked into negative chicken with a concentration of 1000 μg/kg. After the sample pretreatment, the extract solution was measured by ELISA and LFIA.

#### 2.5.3. Spiked and Recovery Test

DNC at different concentrations was spiked into negative chicken. The recovery ratio was calculated according to Formula (1).

Recovery (%) = Concentration Measured/Concentration Spiked × 100%,(1)

## 3. Results and Discussion

### 3.1. Assay Principle of icELISA and LFIA

A schematic illustration of icELISA and LFIA for DNC detection is shown in Scheme 1. DNC (or sample) and mAb solution were added to the ELISA plate, sequentially. The mAb competitively bound to the DNC and coating antigen, then the free compound of antibody–DNC was washed. Subsequently, the HRP-IgG was added and then combined with the mAb. The substrate turned out to be a yellow product under the catalysis of HRP-IgG and the stop effect of H_2_SO_4_. Thus, the substrate solution changed from dark yellow to light yellow with the increased concentration of DNC (Scheme 1a). For LFIA, the NDC solution (or sample) was mixed with the AuNPs–mAb conjugate and then dropped onto the sample pad. The AuNPs–mAb competitively bound to the DNC and coating antigen. Under capillary chromatography, the free AuNPs–mAb combined with coating antigen and formed a T line with red color; the complex of AuNPs–mAb and DNC reacted with goat anti-mouse IgG and formed a control line with red color. The color of the T line changed from dark red (negative) to light red/tintless (positive) with the increased concentration of DNC (Scheme 1b).

### 3.2. Development and Optimization of icELISA for DNC

The parameters for optimizing icELISA were mainly evaluated by optical density (OD) values, inhibition ratio, IC_50_, and A_max_/IC_50_ values. Generally, the conditions corresponding to the moderate A_max_ (the OD value in the absence of DNC, 1.2–1.5), lower IC_50_ value, higher inhibition ratio, and A_max_/IC_50_ values were considered the optimal conditions.

**Figure 1:**
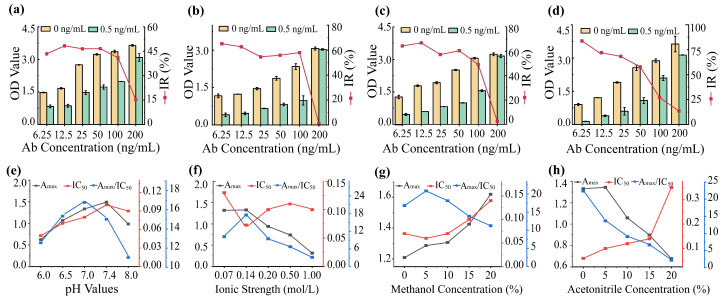
Optimization of icELISA. The concentrations of coating antigen were (**a**) 0.4 µg/mL, (**b**) 0.2 µg/mL, (**c**) 0.1 µg/mL, and (**d**) 0.05 µg/mL with Ab concentrations ranging from 6.25 to 200 ng/mL. The optimization of (**e**) pH values, (**f**) ionic strength, (**g**) methanol concentration, and (**h**) acetonitrile concentration (*n* = 3). Ag indicates coating antigen, Ab indicates mAb, and IR is the abbreviation of inhibition ratio. The yellow and green bar chart in (**a**–**d**) shows the OD value in the presence of DNC at 0 and 0.5 ng/mL, respectively. The error for all graphs was within the standard deviation.

Concentrations of coating antigen and mAb were closely related to the sensitivity of icELISA, and thus, these parameters were primarily analyzed in the conventional icELISA [19]. The OD value rose gradually with the increase in mAb concentration, and the inhibition ratio slowly fluctuated and then decreased, once the coating antigen concentration was given (Figure 1a–d). Higher inhibition ratios were found if the concentration of mAb was 6.25 ng/mL (Figure 1d). In particular, the inhibition ratio was the highest (83.3%) when the mAb and coating antigen concentrations were 6.25 ng/mL and 0.05 μg/mL, respectively, but the OD value in the absence of DNC (A_max_) was lower than 1.2. We abandoned the above concentrations as the optimal condition because of the poor stability of the OD value. By contrast, the satisfied inhibition ratio and OD value were observed at the concentrations of 12.5 ng/mL and 0.05 μg/mL for the mAb and coating antigen.

The pH value and ionic strength of the assay buffer influenced the affinity of the mAb and antigen, which in turn affected the sensitivity of icELISA [20]. First, PBS buffers at different pH values (6.0, 6.5, 7.0, 7.4, and 8.0) were prepared and acted as the assay buffer. Then, its effect on the icELISA was investigated. As shown in Figure 1e, the IC_50_ was the lowest (0.05 ng/mL) at the pH value of 6.0; however, the A_max_ and A_max_/IC_50_ were 0.6 and 12.5, respectively. Actually, it was not enough to comprehensively express the sensitivity of icELISA only by IC_50_ value. The A_max_ value was 1.33, and the IC_50_ value was low, which generated the largest A_max_/IC_50_ value at the pH of 7.0. Different concentrations of ionic strength (PBS, pH 7.0) were prepared, ranging from 0.07 to 1.00 mol/L. A significant decrease in A_max_ was observed when ionic strength varied from 0.14 to 1.00 mol/L (Figure 1f). This phenomenon was mainly caused by the fact that ionic strength at higher concentrations interfered with the formation of hydrogen bonds and reduced the affinity between the mAb and antigen. The A_max_ and A_max_/IC_50_ were the highest and presented an obvious fluctuation at the ionic strength of 0.14 mol/L; thus, the optimal pH value and ionic strength for PBS were 7.0 and 0.14 mol/L, respectively.

As reported, an assay buffer containing organic solvents may contribute to the solubility of the analyte and enhance the formation of hydrogen bonds between the mAb and antigen [21]. Moreover, methanol and acetonitrile are common organic solvents for sample pretreatment. Thus, it was necessary to investigate the tolerance of organic solvents for icELISA. In this study, the IC_50_ values decreased first and then increased as the methanol content increased, and were the lowest at the concentration of 5% methanol (Figure 1g). Meanwhile, the A_max_ increased gradually with the methanol concentration ranging from 0 to 20%. By contrast, the A_max_ decreased when the acetonitrile concentration was higher than 5% (Figure 1h), which indicated the mAb hardly tolerated acetonitrile. Hence, methanol was selected as the extracting solvent, and 5% methanol was a superior concentration for improving the sensitivity of the icELISA.

**Figure 2:**
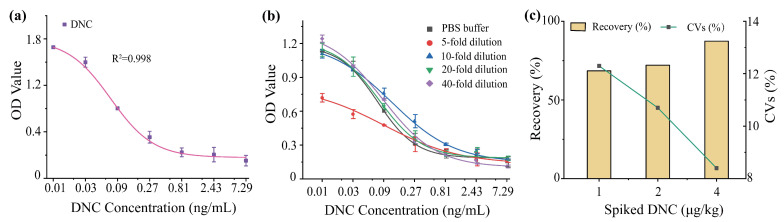
Standard curves for DNC in (**a**) optimal buffer solution and (**b**) matrix solution. (**c**) The recovery ratio and coefficient of variation for the spiked chicken (*n* = 3). CVs was the abbreviation for the coefficient of variation. The error for all graphs was within the standard deviation.

Under the above optimal conditions, a standard curve in the buffer solution was generated with the increase of DNC concentrations (Figure 2a). A four-parameter equation was adopted for the fitting and analysis of the standard curves; the IC_50_ was as low as 0.06 ng/mL and the IC_10_ (limit of detection) was 0.02 ng/mL in the buffer solution.

### 3.3. Characterization of AuNPs and the AuNPs–mAb Conjugate

**Figure 3:**
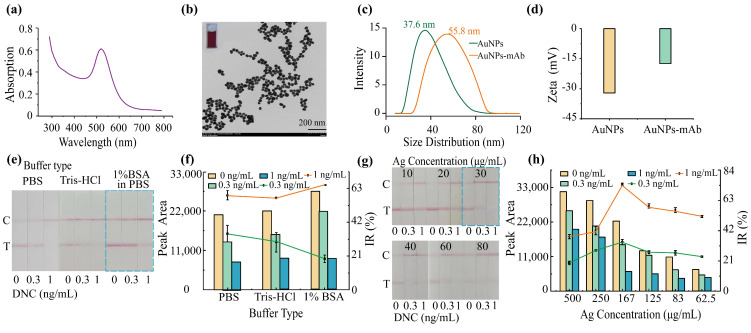
The (**a**) UV−vis spectra of AuNPs; (**b**) the transmission electron micrograph of AuNPs; (**c**) the size distribution of AuNPs and AuNPs–mAb; (**d**) the zeta potential of AuNPs; photographs of test strips optimizing the (**e**) buffer type of mAb and (**g**) Ag concentration, peak area, and IR optimizing the (**f**) buffer type of mAb and (**h**) Ag concentration. Ag indicates the coating antigen, and IR is the abbreviation for the inhibition ratio. The dashed box on the test strips presents the optimal condition. The bar chart in (**f**,**h**) indicates the peak area, and the green and red lines indicate IR in the presence of DNC at 0.3 and 1 ng/mL, respectively. The error for all graphs was within the standard deviation.

The maximum absorption peak of AuNPs was 530 nm measured by the UV–visible spectra (Figure 3a), which corresponded to a size of about 40 nm [22]. As shown in the inserted Figure 3b, the AuNP solution was claret. The transmission electron microscope exhibited that AuNPs appeared elliptic in shape with a diameter of about 40 nm (Figure 3b). Likewise, the dynamic light scattering showed that the size distribution of AuNPs was mainly focused on 37.6 nm (Figure 3c). All the above results illustrated that AuNPs were beneficial for the establishment of LFIA. The size distribution of the AuNPs–mAb was mainly focused on 55.8 nm (Figure 3c). An obvious redshift of the size distribution of AuNPs was observed compared with that for AuNPs, indicating the successful conjugation of AuNPs and mAb. The zeta potentials of AuNPs and the AuNPs–mAb conjugate were −31.5 and −17 mV, respectively (Figure 3d). Compared with the zeta potential of AuNPs, AuNPs–mAb displayed an increased signal, which was dependent on the positive charge of mAb and illustrated the successful conjugation of mAb and AuNPs.

### 3.4. Development and Optimization of LFIA for DNC

In our study, several parameters were optimized to explore the sensitivity of LFIA by the single-factor variable method. The optimal parameters were determined by evaluating the peak area of the T line of the test strip and calculating the inhibition by a two-point (or three-point) competitive format. Generally, the higher peak area and inhibition ratio indicates superior parameters. But there are concrete analyses of concrete conditions.

It has been reported that the antibody conjugated with AuNPs at the highest label efficiency when the AuNP solution was at optimum pH values [23]. In this study, the pH values of the AuNP solution were adjusted by adding various volumes of K_2_CO_3_ (0.1 M). The color intensity of the T line gradually turned darker and then lighter with the increase in the K_2_CO_3_ amounts (Figure S2a). To describe the color intensity of the T line accurately, its peak area was calculated by Image-J software (Figure S2b). The highest peak area was observed when the K_2_CO_3_ amount was 30 µL. Therefore, the optimal pH value of the LFIA was identified when the amount of K_2_CO_3_ was 30 µL. The mAb concentration also played an important role in AuNP–mAb conjugation [24]. As shown in Figure S3a, with the increase in antibody concentration, the color intensity of the T line became darker and then remained balanced in the absence of DNC (negative control). This phenomenon could be explained by the fact that the mAb adsorbed on the surface of AuNPs increased gradually until saturation. The color intensity of the T line turned out to be shallow in the presence of DNC (0.5 ng/mL), which was then assessed by inhibition ratio. However, the unbound mAb remained in the conjugation solution when mAb was at a higher concentration, which resulted in lower inhibition. Obviously, the highest peak area of the T line and inhibition ratio was observed (Figure S3b), and thus, the optimized mAb concentration was 5 μg/mL.

We found that the color intensity of the T line was still weak and appeared to have uneven coloration under the optimized pH values and Ab concentration. We conjecture that the dilution buffer solution (PBS, 20 mM, pH 7.4) of mAb might be not suitable for the labeling procedure of LFIA. Hence, we compared the effects of three different buffer types on the color intensity and sensitivity for LFIA. As shown in Figure 3e, the negative control of the T line showed the darkest color when the buffer solution was 1% BSA solution (in PBS, 20 mM, pH 7.4). By contrast, Tris-HCl showed the lightest color of the T line. We believe that BSA can improve the dispersion and stability of the AuNPs–mAb conjugate, as well as the color intensity of the T line. The highest peak area of the T line was observed when the buffer type was 1% BSA (Figure 3f). At this point, the inhibition ratio was 20% in the presence of DNC at 0.3 ng/mL, while it was the highest (65%) in the presence of DNC at 1 ng/mL. The 1% BSA was finally selected as the best buffer type, considering that the color intensity was related to the stability of LFIA.

With the increase in the AuNPs–mAb amount, the color intensity of the T line for negative control became gradually deeper (Figure S4a). The color intensity of the T line was evident when the amount of AuNPs–mAb added was 4 and 5 μL. But its corresponding inhibition ratio was low even if the DNC concentration was at 0.3 or 1 ng/mL (Figure S4b). Despite displaying a higher inhibition ratio for a 2 μL AuNPs–mAb amount when the DNC concentration was 0.3 ng/mL, the inhibition ratio was inconspicuous at a higher DNC concentration (1 ng/mL), and the color intensity was low. Thus, the optimal AuNPs–mAb amount was 3 μL.

**Figure 4:**
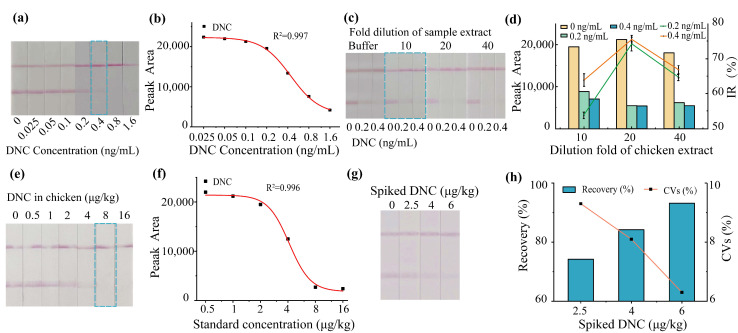
(**a**) Photographs of test strips and (**b**) standard curve with the increasing DNC concentrations in buffer solution. (**c**) Photographs of test strips, (**d**) peak area, and IR when exploring the matrix effect. (**e**) Photographs of test strips and (**f**) standard curve with the increasing DNC concentrations in chicken. (**g**) Photographs of test strips and the (**h**) recovery test in the spiked chicken. The bar chart in (**d**) shows the peak area and the green and red lines indicate IR in the presence of DNC at 0.2 and 0.4 ng/mL, respectively. The error for all graphs was within the standard deviation.

The concentration of coating antigen also played a key role in the establishment of LFIA, and its evaluation method was similar to the above analysis method. It was observed that the higher the coating antigen concentration, the deeper the color intensity of the T line, and this led to a decrease in sensitivity (Figure 3g). A dynamic increase in inhibition ratio was recorded when the concentration of coating antigen was 167 μg/mL (Figure 3h). Thus, the comprehensive consideration of combining both photographs and quantitative analysis was necessary for the establishment of LFIA. Finally, the preferred concentration of coating antigen was 167 μg/mL. Under the optimal conditions, a standard curve was generated with the increase in DNC concentrations (Figure 4a, b). In the buffer solution, the visual limit of detection (cut-off value) and quantitative limit of detection were 0.4 and 0.16 ng/mL, respectively.

### 3.5. Sensitivity, Specificity, and Accuracy of icELISA and LFIA in Chicken

We adopt a simple sample pretreatment method to save testing time in this study. In detail, the chicken was extracted with methanol, and the extraction was diluted with a buffer solution, aiming to eliminate the matrix effect. Generally, the standard curve in diluted extraction matches well with that in the buffer solution, which means the matrix effect could be eliminated [25].

As shown in Figure 2b, a serious matrix effect was observed at the 5-fold dilution, which implied that the matrix components interfere with the affinity between mAb and the antigen. The effect of the matrix on the sensitivity of icELISA decreased as the dilution factor increased. The standard curves in the matrix solution were almost consistent with that in the buffer solution when the dilution factor was higher than 20. Thus, a 20-fold dilution was adopted as the preferred dilution factor. According to the matrix standard curve, the limit of detection for icELISA was 0.8 μg/kg in chicken.

With the increase in the dilution fold of extraction, the color intensity of the T line showed almost the same when the DNC concentration was 0 ng/mL (Figure 4c); however, the inhibition ratio exhibited obvious superiority (Figure 4d). Although a higher inhibition ratio was revealed, the limit of detection was worse due to the higher dilution fold. The inhibition ratio at 0.2 and 0.4 ng/mL in the 10-fold dilution was similar to that in the buffer solution, which meant that a 10-fold dilution could eliminate the matrix effect. After the sample pretreatment, the matrix standard curve was generated, which showed that the visual limit of detection (cut-off value) and quantitative limit of detection were 8 and 2.5 μg/kg, respectively (Figure 4e,f).

We systematically investigated the sample pretreatment of icELISA and LFIA. It was observed that a 10-fold dilution of sample extract solution could eliminate the matrix effect of chicken for LFIA; however, a double dilution fold was required for icELISA. Although the same mAb and coating antigen were applied for both icELISA and LFIA, the LFIA exhibited higher matrix tolerance to the sample extract of chicken. We considered that the difference in mAb and coating antigen concentrations and the activity of horseradish peroxidase influenced the matrix effect. The higher concentration of mAb and coating antigen might improve the matrix tolerance of LFIA. Despite possessing higher sensitivity for icELISA, the trace concentration of mAb and coating antigen was more likely to be interfered with by the matrix factor. Moreover, the optical density of icELISA was produced by horseradish peroxidase catalyzing tetramethylbenzidine. The activity of horseradish peroxidase was significantly interfered with by the matrix factor [26], and a higher dilution fold was required to eliminate the matrix effect.

To decrease the matrix effect of samples, some researchers have tried different sample pretreatments (Table 1). Xu et al. selected methanol (containing 1% acetic acid, 15 mL) as the extract solution, and adopted centrifugation to separate the supernatant, which was then blow-dried under nitrogen. Then, the extract was dissolved in phosphate-buffered solution (3 mL) followed by a measurement using ELISA and LFIA [27]. Compared with the more complex sample pretreatments [28,29], some simple attempts have also been reported. Zhang and Xu et al. used the procedure of extraction and direct dilution to decrease the interference of chicken [30]; however, the matrix effect for a different dilution factor of the extract was not shown in detail. In this study, we investigated the matrix effect of chicken professionally and developed a simple sample pretreatment method for the development of icELISA and LFIA. Although the dilution factor of the extract solution was not the lowest compared with other studies, the sensitivity could still satisfy the requirement of detection, and the stability was excellent.

As reported, the immunoassay usually has the advantage of high specificity, because of the precise recognition of mAb and antigen [31]. Here, three veterinary medicines (including chloramphenicol, trimethoprim, and sulphanilamide) at 1000 μg/kg were spiked into chicken, and then the recoveries of icELISA and LFIA were measured. Specifically, the cross-reactivity was lower than 0.01% and 0.8% for icELISA and LFIA (Figures S5 and S6). The negligible cross-reactivity illustrated the high specificity of both icELISA and LFIA. The accuracy of the developed immunoassay was detected by the spiked and recovery test. The negative chicken sample was spiked into DNC at the concentrations of 1, 2, and 4 μg/kg, and then measured by the icELISA developed in this study. As displayed in Figure 2c, the recovery ratio ranged from 68.5% to 87.4% with a coefficient of variation of 8.4–12.3%. Likewise, the DNC at the concentrations of 2.5, 4, and 6 μg/kg was added in the negative chicken sample, and then detected by the LFIA developed in this study. The results showed that the recovery ratio ranged from 74.2% to 93.2% with a coefficient of variation of 6.3–9.3%. Therefore, the icELISA and LFIA established in this study maintained good performance in accuracy and stability, and are thus reliable analytical methods for the detection of DNC.

## 4. Conclusions

In summary, the comparable icELISA and LFIA were developed as efficient and straightforward tools for DNC screening in chicken. Owing to the signal amplification of horseradish peroxidase-catalyzed tetramethylbenzidine, the icELISA exhibited relatively high sensitivity (at least eightfold) compared to LFIA; however, it also displayed low tolerance of the chicken matrix using the same sample pretreatment procedure. After sample pretreatment, the limit of detection of the icELISA for DNC was calculated to be 0.8 μg/kg with an average recovery of 68.5–87.4% and a coefficient of variation of less than 12.3%. The visual and quantitative limits of detection of the LFIA for DNC were 8 and 2.5 μg/kg, which could be detected within 15 min. Meanwhile, the average recovery of the LFIA ranged from 74.2% to 93.2%, and the coefficient of variation was less than 9.3%. Overall, the established icELISA and LFIA met the requirement of rapid detection in terms of sensitivity, specificity, and accuracy. They also provided mutually verifiable assays for the detection of DNC in samples, which could improve the accuracy of the detection results.

## Figures and Tables

**Table 1:** An overview and comparison of the immunoassays for the detection of DNC.

Strategy	Samples	LOD ^a^ (µg/kg or µg/L)	Dilution Factor	Recovery (%)	Extraction Procedure	References
ELISA	Egg and chicken	0.3	10	71–113.6	Extract solvent: acetonitrile; extract supernatant diluted with PBS.	Zhang et al., 2023 [30]
ELISA	Chicken/duck	- ^b^	150	74.4–111.7	Extract solvent: methanol; extract supernatant was diluted.	Shen et al., 2022 [14]
ELISA	Egg	2.2	2	80.8	Extract solvent: acetonitrile; extract supernatant was evaporated to dryness; hexane and methanol/water was added to remove fat; evaporated to dryness again; residues were reconstituted with methanol/water.	Bilandzic et al., 2013 [29]
ELISA	Chicken	10	-	67	Extract solvent: acetonitrile; extract supernatant was evaporated to dryness; hexane and methanol/water was added; evaporated to dryness again; residues were reconstituted with 5% methanol.	Huet et al., 2005 [28]
	Egg	3		85		
ELISA	Chicken/pork/beef	50	40	75–95	Extract solvent: acetonitrile; extract supernatant was evaporated to dryness; the extraction was dissolved in sample diluent.	EAGEN™
LFIA	Chicken	2.5	-	93.5–115.6	Extract solvent: methanol containing 1% acetic acid; extract supernatant was evaporated to dryness and then dissolved in PBST solution.	Xu et al., 2020 [27]
	Liver	5		99.6–108.0		
LFIA	Milk	100	-	99–100	No sample pretreatment.	Wu et al., 2018 [13]
FPIA ^c^	Chicken	24.21	5	74.2–80.8	Extract solvent: methanol; the supernatant was diluted with an assay buffer.	Zhang et al., 2021 [25]
Time-resolved fluorometry	Egg	3.2	50	97.3–115.6	Extract solvent: acetonitrile; extract supernatant was evaporated to dryness; methanol/deionized water reconstituted dried samples.	Hagren et al., 2004 [9]
	Liver	11.3	100	98.4–103.6	Extract solvent: acetonitrile; extract supernatant was evaporated to dryness and continued with a hexane wash; methanol/deionized water reconstituted dried samples; mixture solution was evaporated to dryness, methanol/deionized water reconstituted dried samples.	
ELISA	Chicken	0.8	20	68.5–87.4	Methanol was the extract solvent; the supernatant was diluted with an assay buffer.	This study
LFIA		8–2.5 ^d^	10	74.2–93.2		

^a^ indicates the limit of detection. ^b^ not shown exactly in the paper. ^c^ is the abbreviation for fluorescence polarization immunoassay. ^d^ indicates that the visual and quantitative limits of detection of LFIA were 8 and 2.5 μg/kg.

## Data Availability

All data are contained within the article or Supplementary Material.

## Supplementary Materials

The following supporting information can be downloaded at: https://www.mdpi.com/article/10.3390/toxics11070628/s1, Supplementary Materials: Figure S1: Chemical structures of 4,4′-dinitrocarbanilide and 2-hydroxy-4,6-dinitrocarbanilide; Figure S2: Optimization of K_2_CO_3_ amounts for LFIA; Figure S3: Optimization of mAb concentration for LFIA; Figure S4: Optimization of the amount of AuNPs–mAb for LFIA; Figure S5: The cross-reactivity for icELISA; Figure S6: The cross-reactivity for LFIA.
