# Supplementary material for: A Comparable icELISA and Lateral Flow Immunoassay for the Sensitive and Rapid Detection of 4,4′-Dinitrocarbanilide in Chicken
Source: Toxics. 2023 Jul 20;11(7):628. doi: 10.3390/toxics11070628 (PMC10385411; doi:10.3390/toxics11070628)
Supplement: Supplementary file 1 [file toxics-11-00628-s001.zip › toxics-2483918-supplementary.pdf]

Figure Captions

Figure S1. Chemical structures of 4,4'-dinitrocarbanilide and 2-hydroxy-4,6-dinitrocarbanilide.

Figure S2. Optimization of K_2CO_3 amounts for LFIA.

Figure S3. Optimization of mAb concentration for LFIA.

Figure S4. Optimization of the amount of AuNPs-mAb for LFIA.

Figure S5. The cross-reactivity for icELISA.

Figure S6. The cross-reactivity for LFIA.

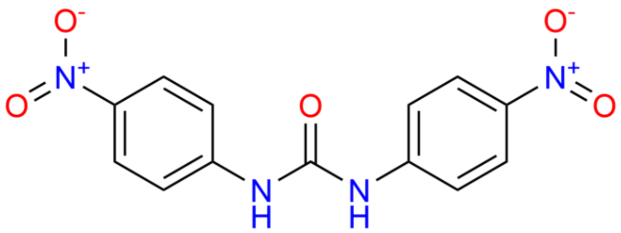

4,4'-dinitrocarbanilide

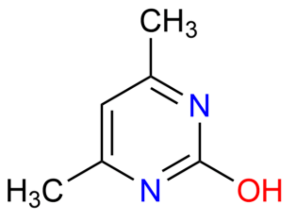

2-hydroxy-4,6-dinitrocarbanilide

Figure S1. Chemical structures of 4,4'-dinitrocarbanilide and 2-hydroxy-4,6-dinitrocarbanilide.

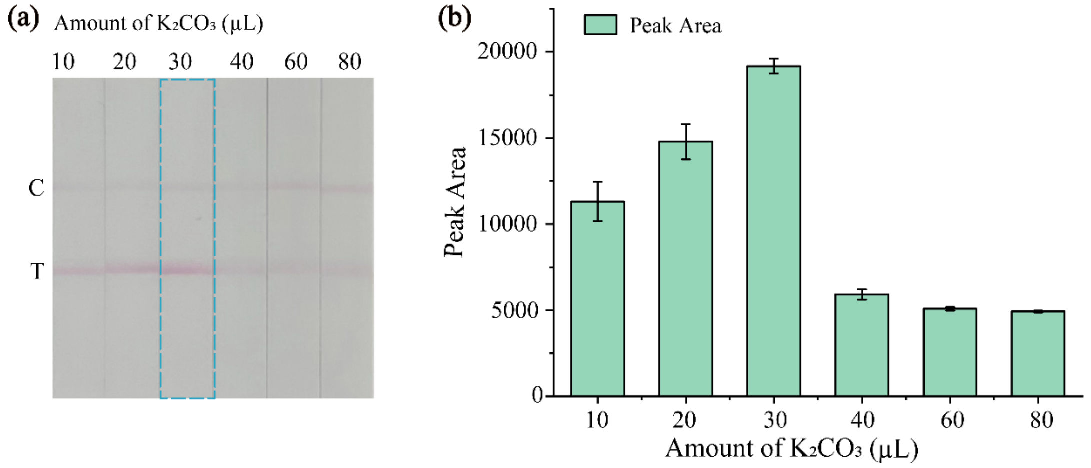

Figure S2. Optimization of K_2CO_3 amounts for LFIA. (a) Photographs of test strips and (b) peak area of T line with the increasing of K_2CO_3 amount. The dashed box on the test strips presented the optimal condition.

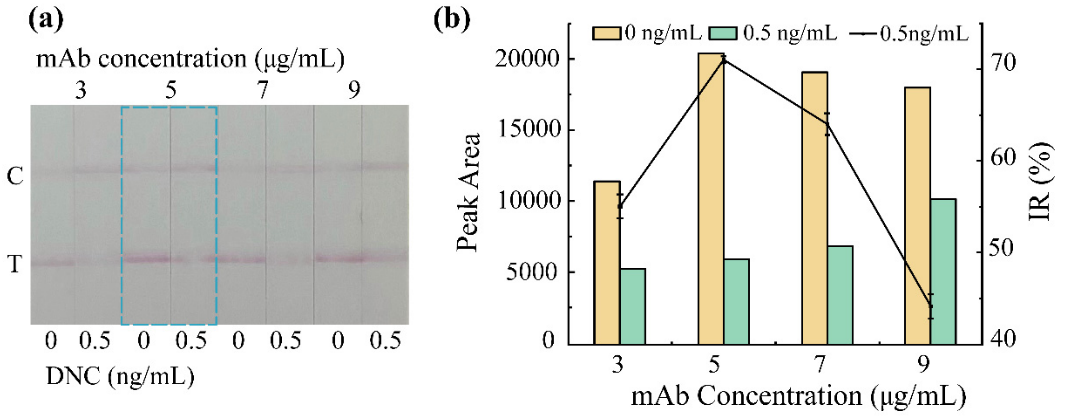

Figure S3. Optimization of mAb concentration for LFIA. (a) Photographs of test strips and (b) peak area of T line in the present (0.5 ng/mL) or absent (0 ng/mL) of DNC with the increasing of mAb concentration.

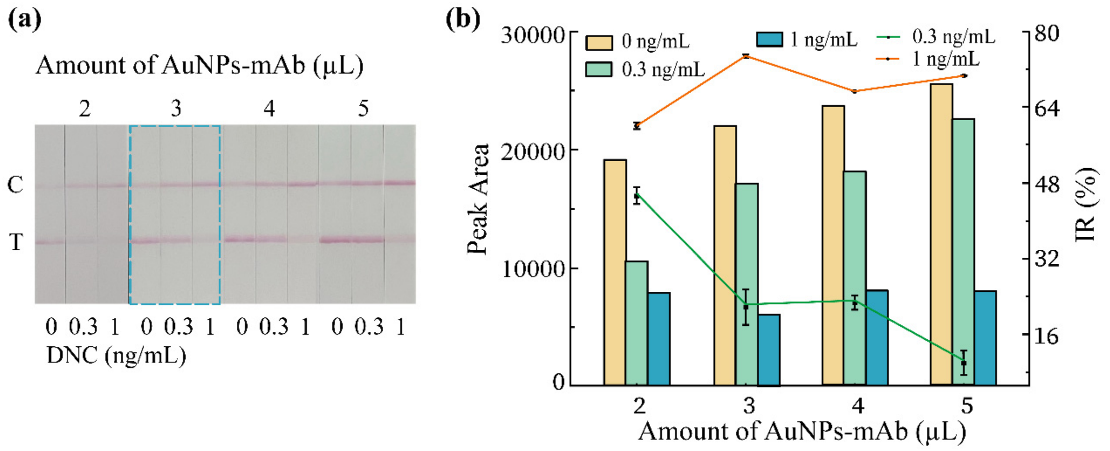

Figure S4. Optimization of the amount of AuNPs-mAb for LFIA. (a) Photographs of test strips and (b) peak area of T line in the present (0.5 and 1 ng/mL) or absent (0 ng/mL) of DNC with the increasing of the amount of AuNPs-mAb.

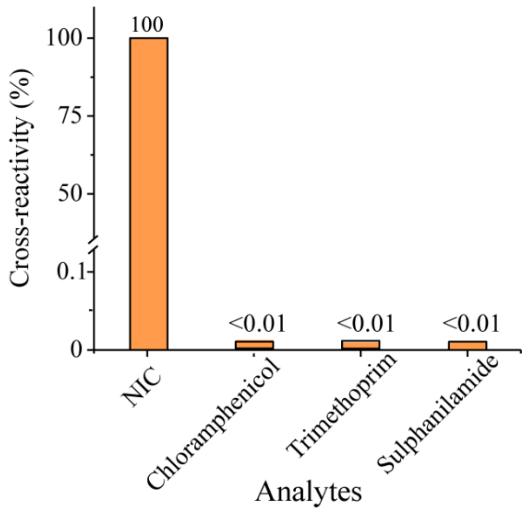

Figure S5. The cross-reactivity for icELISA against DNC, chloramphenicol, trimethoprim, and sulphanilamide. The analytes were spiked in chicken with a concentration of 1000 μg/kg, except for DNC (1.0 μg/kg).

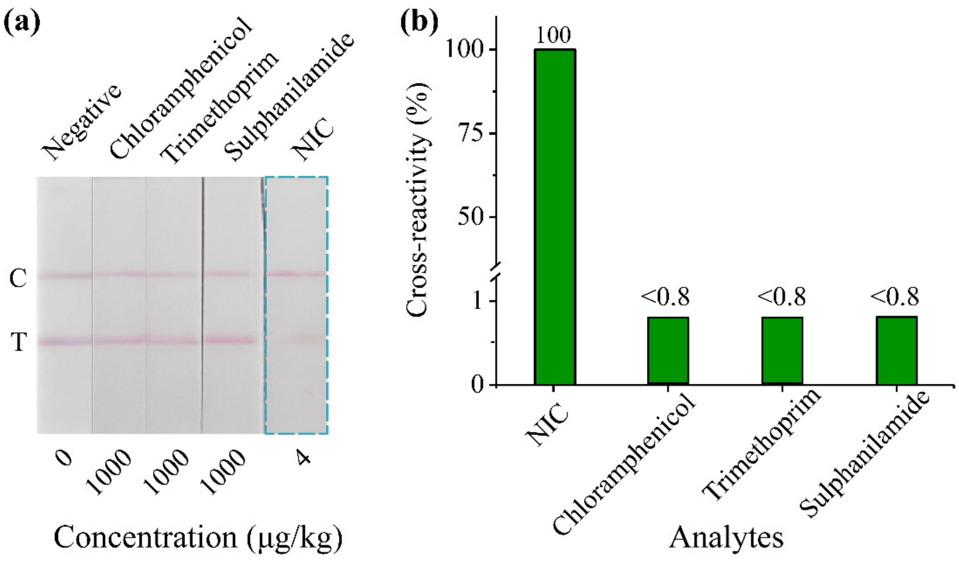

Figure S6. The specificity for LFIA. (a) Photographs of test strips and (b) cross-reactivity against DNC, chloramphenicol, trimethoprim, and sulphanilamide. The analytes were spiked in chicken with a concentration of 1000 μg/kg, except for DNC (8 μg/kg).
